# Relationship between aerobic fitness and metabolic power metrics in elite male soccer players

**DOI:** 10.5114/biolsport.2022.106389

**Published:** 2021-07-28

**Authors:** Vincenzo Manzi, Giuseppe Annino, Cristian Savoia, Giuseppe Caminiti, Elvira Padua, Matteo Masucci, Rosario D’Onofrio, Ferdinando Iellamo

**Affiliations:** 1 Dipartimento di Scienze Umanistiche, Università Telematica Pegaso, Naples 80132, Italy; 2 Dipartimento di Medicina dei Sistemi, Università Tor Vergata, Roma, Italy; 3 Research Institute for Sport and Exercise Sciences, The Tom Reilly Building, Liverpool John Moores University, Liverpool, UK; 4 Istituto di Ricovero e Cura a Carattere Scientifico San Raffaele Pisana, Roma, Italy; 5 Department of Human Science and Promotion of Quality of Life, San Raffaele Open University of Rome, 00166 Rome, Italy; 6 University of Waterloo, Faculty of Kinesiology, Iowa, USA; 7 Scientific Society of Sport Rehabilitation and Posturology, Lazio, Italy; 8 Scuola di Specializzazione in Medicina dello Sport e dell’Esercizio Fisico, Dipartimento di Scienze Cliniche e Medicina Traslazionale, Università Tor Vergata, Roma

**Keywords:** Anaerobic threshold, Video match analysis, Kinematic parameters, Metabolic power, Training methodology

## Abstract

The aim was to assess the relationship between aerobic fitness and metabolic power metrics in elite male soccer players, and the possible differences that playing positions might impose during match play over new metabolic power metrics. Sixty-two elite professional male soccer players (13 central backs, 13 side backs, 22 midfielders, and 14 forwards) took part in the study. Players were monitored during eleven months of full training (including pre-season and in-season) and over all official matches (Serie A matches, Italy Cup matches). Aerobic fitness tests were conducted one week after the start of the preseason, and 8, 24 and 36 weeks after the beginning of the Championship. Players’ aerobic fitness and metabolic power metrics were considered as the mean of all seasonal testing and of pooling data of 38 championship matches and 3 or 6 Italy Cup matches for all the calculations respectively. The velocity at 4 mmol·L^-1^ (VL_4_) was significantly related to metabolic power metrics match variables with correlation ranging from trivial to very large (r = 0.32 to r = 0.89). Receiver-operating-characteristic (ROC) analysis showed that speed at VL_4_ was sensitive in detecting high metabolic power distance (HMPD) changes in all but central back players as revealed by area under the curve (central back .78, 95%CI .47 to .95; full back .93, 95%CI .64 to 0.99; midfielder .88, 95%CI .67 to 0.98; forward .90, 95%CI .62 to 0.99). This study’s findings provide further evidence for the ecological validity of aerobic fitness in elite male soccer players. Players having a HMPD cut-off equal to or higher than > 1450 m for central backs, > 1990 m for full backs, > 2170 m for midfielders and > 1670 m for forwards may be considered as possessing superior aerobic fitness status. In light of this study’s findings, the VL_4_ test may be considered a valid test to evaluate meaningful information for direct generic aerobic training in soccer players.

## INTRODUCTION

The execution of soccer-related movements imposes large physiological loads on players during competition. Consequently, the players’ metabolic pathway is heavily stressed to meet physical and physiological demands during the official match. Elite players perform at 85–90% of their maximal heart rate, with an average oxygen uptake (V̇O_2_) 70–80% of the maximum [1]. Despite a low average match speed (110–140 m·min^-1^, in a 90-minute game), soccer players may experience remarkable neuromuscular fatigue and energy expenditure during the game [2]. Indeed, the intermittent nature of a soccer match imposes upon elite players, during a 90-minute game, 1,200–1,400 activity changes and 150–200 short multi-directional high-intensity efforts (1–6 seconds). [1, 3].

These changes in movement patterns can only be performed by providing players with high physical conditioning. In this regard, the most recent research stressed the importance of the development of aerobic and anaerobic metabolism and muscular power as a prerequisite for playing soccer successfully, given that high intensity is associated with the most decisive events in a soccer game [4, 5]. Usually, a V̇O_2max_ of 60 ml·kg^-1^·min^-1^ was recommended as the minimum aerobic power requirement to successfully play soccer at a professional level in male athletes [1].

In recent years, the advances in player tracking technology (e.g., Global Positioning System – GPS, semi-automated computerized tracking systems) allowed researchers to estimate many kinematic and physiological parameters during matches and training sessions, that could not be analysed in the past (i.e., acceleration, deceleration, overall energy expenditure) [6]. Before the advances in technology researchers could only measure the distance and the time travelled at different running speeds [7]. The speed category approach has been found to provide partial knowledge of actual game physiological demands, since it does not take into account kinematic aspects such as acceleration and deceleration. Both of these parameters affect a player’s overall energy expenditure during a game or a training session [6, 8]. Recently, in an attempt to describe the detailed kinematics and metabolic demands during competition or training, a new method defined as metabolic power was proposed by Osgnach et al. [6]. The Osgnach metabolic power computation represents a synthetic index of the physical work (per unit of time) and constitutes an integrated method that is useful to assess both aerobic and anaerobic energy expenditure of players during both the match play and training. With such a metabolic power-based method, the energy expenditure during soccer match play has been proved to be underestimated in comparison to traditional measurement of running speed alone, since high energy demand can be imposed on players even when speed is low, but the acceleration is high [6, 9]. Indeed, using the metabolic power based method, anaerobic contributions greater than those previously observed with the speed category approach were reported. Research related to metabolic power metrics is limited, along with its association with a soccer players’ physiology. To date, only one study has examined the relationship between aerobic fitness and metabolic power metrics in an official match. The authors provided in this study descriptive evidence of an association between aerobic fitness and match metabolic power categories [10]. Recently, it was proposed to analyse high-intensity activity profiles of elite soccer players using a new algorithm in the metabolic power metrics that can detect the number of “power events” during a game or training session [9]. The metabolic power events (MPE) are high intensity bouts that are computed by plotting together the time course of the estimated metabolic power and the oxygen consumption. MPE identifies when the player “pushes on the gas pedal”, regardless of the speed at which he is. It corresponds to all those events with a high energy demand (anaerobic), which subsequently need a recovery time (to pay the owed oxygen debt) [9].

It has been argued that, instead of the distance covered in high-power categories, power event metrics could provide better and more valid information of high-intensity physical demands in official matches and training and they would therefore be worth exploring [9, 11]. Information on the association between aerobic fitness and power events during an official match could make it possible to set up interventions during training that better reflect actual match play demands [9, 11].

Therefore, the aim of this study was to examine the associations between aerobic fitness – as measured using standard laboratory tests – and match power metrics in male professional soccer players and the possible differences that different playing positions might impose during match play over new metabolic power metrics. The existence of association between aerobic fitness and selected measures of physical performance during actual match play was assumed as the work hypothesis

## MATERIALS AND METHODS

### Experimental Approach to the Problem

The metabolic power and the kinematic parameters associated with it constitute an integrated measure of acceleration and velocity, and better estimate the physiological effort of the soccer players during actual match play or training [6].

The results of a previous study showed that players’ external load – the distance covered in arbitrary chosen energy-expenditure categories, expressed as metabolic power – was strongly to very strongly correlated with all aerobic fitness variables (i.e., V̇O_2max_, V̇O_2_VT, VL_4_) [12].

In fact, this study provided information that aerobic fitness is an essential performance component in male professional soccer as per its convergent construct validity with match metabolic power variables.

However, in the study by Manzi et al. the research design with convenience sampling (i.e., team study) and the small cohort size limit the external validity and consequently the study result generalization [10]. A practical strategy to solve this design bias maintaining group focus (i.e., elite level) in an ecological setup is inductive evidence. This was done with study replication under environmental (ecological setup) and experimental (i.e., similar independent variables) analogous conditions [13].

Therefore, this research was carried out with the aim to provide further evidence that aerobic fitness (e.g., submaximal aerobic fitness variables such as velocity at selected blood lactate concentrations) is a relevant performance component in male professional soccer players [14]. This descriptive, non-experimental, multi-centre study was implemented during pre-season and in-season for four professional soccer teams (i.e., Italian Serie A). In this study, relationships between variables were performed by pooling data of 38 championship matches and 3 or 6 Italy Cup matches, in which players were observed from a minimum of 6 to a maximum of 32 times. Goalkeepers and players with fewer than 6 records were not included in the analysis. The running speed at a blood lactate concentration of 4 mmol·L^-1^ (VL_4_) – based on the result of treadmill testing – was assumed as a physiological paradigm for aerobic fitness [13, 15]. This testing procedure was used because a number of soccer studies have reported greater sensitivity of submaximal aerobic fitness variables in tracking seasonal changes in endurance performance than in V̇O_2max_ and a major relationship with match performance [13, 15, 16, 17]. Furthermore, enhancements in submaximal aerobic fitness variables positively affected the amount of high-intensity exercise accumulated during matches in soccer players [10].

The latter association may be considered as an added value for using submaximal tests during the preparatory and competitive season in professional soccer. With the aim of fostering data consistency, players’ aerobic fitness was considered as the mean of three or four seasonal assessments [10]. Particularly, tests were conducted one week after the start of the preseason, and 8, 24 and 36 weeks after the beginning of the Championship. Match activities were classified according to the procedures suggested by Osgnach et al. and expressed as high metabolic power distance (HMPD) (> 25 W·kg^-1^), number of power events (n), average duration of power events (s), power events average power (W·kg^-1^), average power recovery between power events (W·kg^-1^) and recovery time between power events (s) [9].

Power events are metabolic power metrics that are dependent on anaerobic processes that mainly reflect high intensity match activities, calculated by plotting together the time course of estimated metabolic power and oxygen consumption [9]. It is assumed that HMPD, the number of power events and recovery time between power events (i.e., repeated high-intensity sequences) were the reflection of activities leading to actions relevant to match outcome [6, 11, 18].

A cut-off value of 25 W·kg^-1^ was chosen according to Martín-García et al. [18]. This metabolic power value was considered as corresponding to a velocity of 19 km·h^-1^, thus in line with soccer players’ very high intensity activity [10]. Additionally, to establish the effectiveness of aerobic fitness to better maintain the spatial structure of the team, we examined its relationship with the average power recovery and the average time recovery after high-intensity bouts [18].

In this regard, it could be speculated, given the intermittent nature of soccer, that there is a superior average power recovery between high-intensity bouts in those players possessing greater aerobic fitness. Given the supposed ability of the metabolic power approach to provide a more detailed profile of players’ match physical performance, no information deriving only from velocity was used in this research analysis. To limit possible occurrence of type I errors, a pre-planned comparison approach addressing the association between aerobic fitness and metabolic power metrics was used [19]. It is assumed that metabolic power metrics were the reflection of activities leading to the most decisive events in a soccer game [1, 3, 6, 10]. Descriptive statistics and analysis were then calculated based on playing position. These data were then averaged across all observations per position for between-group analysis.

### Subjects

This study included 62 male professional Italian Serie A soccer players. Characteristics of players are reported in Table 1A. All the players were active members of 4 different squads of the Serie A championship. Players were grouped according to their playing position as central backs (CB, n = 13), full backs (FB, n = 13), midfielders (MF, n = 22), and forwards (FW, n = 14). Players had at least 5 years of competitive experience in the premiership and were monitored over a whole season (11 months) including pre-season, in-season, and all official matches (Serie A matches, Italy Cup matches) which took place from July until May in the seasons 2014–15, 2015–2016, 2016–2017, 2018–2019. The squads systematically played in a 4-3-3 formation model with four defenders (two FBs and two CBs), three midfielders (MFs) and three forwards (FWs). All players underwent the same training modality sessions 7 times a week throughout the pre-season with a friendly match played on a Thursday or during the weekend. Training sessions were mainly devoted to technical-tactical skill development with fitness training sessions performed with a single training modality (i.e., no other exercises) during pre-season. During the championship, players trained 6 times per week, with a match played during the weekend. Friendly and cup matches usually occurred on Thursday and Wednesday, respectively. Training volume and intensity were prescribed to players by the same strength and conditioning staff and the same technical-tactical coaches, who moved all together from one team to another over the years (the first author of the study was the team’s fitness coach over the years). Each professional player gave his informed consent about the research purposes of use of the results observed during their usual training sessions and official matches.

**TABLE 1A:** Physiological and metabolic power metrics data according to playing position.

Variables	Central back (n = 13)	Full back (n = 13)	Midfielder (n = 22)	Forward (n = 14)
**Max heart rate (bpm)**	184 ± 8	191 ± 9	189 ± 6	193 ± 11
**Max lactate post treadmill test (mml·L^-1^)**	9.31 ± 2.20	10.16 ± 1.58	9.53 ± 3.20	8.13 ± 2.17
**Velocity at 4 mmol·L^-1^ (km·h^-1^)**	13.82 ± 0.56	14.46 ± 0.69	15.08 ± 0.73^#^	14.24 ± 0.84
**Totale distance (m)**	10018 ± 385	10846 ± 616	11523 ± 650	10230 ± 663
**Anaerobic index (%)**	36.52 ± 3.83	37.07 ± 3.96	34.70 ± 4.31	36.19 ± 4.96
**Equivalent distance (%)**	13.55 ± 1.85	14.92 ± 2.19	14.86 ± 3.04	14.62 ± 2.72
**Distance > 25 Watt·Kg^-1^ (m)**	1475 ± 132	1995 ± 198^§^	2205 ± 289^†^	1783 ± 390^§^
**Power Events (number)**	136 ± 17	149 ± 17	181 ± 24^†*^	144 ± 16
**Power events recovery power (Watt·Kg^-1^)**	5.86 ± 0.80	6.20 ± 0.74	6.75 ± 0.65^†^	5.60 ± 0.60
**Power events recovery time (s)**	30.79 ± 5.02	29.32 ± 4.35	24.09 ± 3.64^#*^	29.28 ± 5.59
**Power events average time (s)**	4.93 ± 0.84	5.31 ± 1.00	5.07 ± 1.13	4.91 ± 1.04
**Power events average power (Watt·Kg^-1^)**	26.15 ± 1.64	27.29 ± 2.00	26.56 ± 1.82	28.12 ± 2.17^§^

Note: Data are reported as mean ± SD;

§ p < 0.05 different from CB;

# p < 0.01 different from FW and CB;

† p < 0.001 different from FW and CB;

* p < 0.05 significantly different from SB.

The study was approved by the local Institutional Review Board before the start of the study. All the procedures involved in this study were in accordance with the Declaration of Helsinki.

### Fitness Assessment and Video Match Analysis

Players performed a 2-phase progressive treadmill test (Technogym Run Race 1400 HC, Gambettola, Italy) for the assessment of individual blood lactate concentration profiles and maximal HR, respectively, on three (11.3% of all players) or four occasions (at the start, after 8, 24 and 36 weeks of training).

The progressive treadmill test consisted of four to six submaximal exercise bouts at an initial running speed of 9 km·h^-1^ and interspersed with 1-min recovery, followed by a maximal incremental test to volitional fatigue. The treadmill running velocity was increased during the submaximal test by 1.5 km·h^-1^ every 4 min. Once capillary blood lactate concentrations were elevated to more than 4 mmol·L^-1^, the treadmill speed was increased 0.5 km·h^-1^ every 30 s until exhaustion as done in previous studies [13, 15]. The mean test duration was 22 ± 4.5 minutes with a range of 9.5 min. Capillary blood samples were taken from the earlobe immediately after each submaximal bout and three minutes after exhaustion and analysed to assess exercise blood lactate concentrations using a portable amperometric microvolume (5 ml) lactate analyser (LactatePro, Arkray, Tokyo, Japan). Before each test, the analyser was calibrated by following the manufacturer’s recommendations. HR was recorded every 5 s with a short-range telemetry system (Polar Team System; Polar Electro Oy, Kempele, Finland) during all assessments. The highest HR measured during the maximal incremental test was used as the maximum reference value (HR_max_). The criteria for HR_max_ achievement were blood lactate concentrations higher than 8 mmol·L^-1^ and HR plateau attainment despite velocity increments. Blood lactate concentrations were plotted against running speeds, and individual blood lactate concentration profiles (velocity at 4 mmol·L^-1^, VL_4_) were identified via exponential interpolation [20]. Potential confounding effects of previous exercise fatigue on variables were minimized by ensuring that coaches refrained their players from heavy training on the day preceding assessments. A record of the nutrient content was taken to provide sufficient carbohydrate intake during the week before assessments. Before, during, and after all testing sessions and games, hydration was promoted allowing “ad libitum” drinking in all players. Throughout the study, all testing sessions took place at the same time of the day (between 9.00 a.m. and 1.00 p.m.) to avoid circadian influences.

The progressive treadmill testing procedure used in the present investigation has been reported as highly reproducible [13, 14, 15]. Match analysis was performed using the validated multicamera video analysis system STATS SportVU [STATS LLC, Chicago (IL), U.S.A.], tracking at rates of up to 25 Hz. The Technical University of Munich (TUM) determined the measurement accuracy of this device with a typical error of 2.7% for total distance [21]. Raw data were provided via cartesian coordinates by K-Sport (primary data have been smoothed at 5 Hz). The STATS SportVU tracking system transports the data of performance by extracting and processing coordinates of players (X, Y) and the ball (X, Y, Z) through HD cameras as well as sophisticated software and statistical algorithms [21]. Through cameras located at roof level, player movements were captured during matches and the data analysed by two sources, STATS Viewer and STATS Dynamix, all offered by STATS Perform to create a dataset on each player’s physical and technical performance. The players analysed and used for subsequent analysis either ended the game or were substituted no more than 5 minutes before the final whistle (≥ 85 min of match play). Data extracted from STATS SportVU were downloaded and analysed using specific software (https://www.gpexe.com). The data considered for analysis included high metabolic power distance (HMPD > 25 W·kg^-1^), number of power events (n), average power recovery between power events (W·Kg^-1^) and other metabolic power metrics. Data were filtered according to the theoretical model based on an energetic approach where the energy cost of accelerations and decelerations plays a central role, as suggested by Osgnach et al. [6].

### Statistical Analysis

The results are expressed as mean ± SDs. Assumption of normality was verified using the Shapiro-Wilk W-test. Variables’ association was assessed using Pearson’s product moment correlation coefficient (r) and provided with the corresponding confidence interval at 95%. Linearity was assumed after visual inspection of variable-associated scatterplots and in case of doubt, comparing r values with eta values. Qualitative magnitude of associations was reported as follows: trivial r < 0.1, small 0.1 < r < 0.3, moderate 0.3 < r < 0.5, large 0.5 < r < 0.7, very large 0.7 < r, 0.9, nearly perfect r < 0.9, and perfect r = 1 [22]. A one-way between-group analysis of variance was conducted to explore whether the group difference influenced each of the physiological and performance variables. Simple main effects were calculated using a Bonferroni correction. Partial eta squared (η^2^) was reported as a measure of effect size [23]. Effect sizes of 0.1, 0.1 to 0.20, 0.20 to 0.50, 0.50 to 0.80, and > 0.80 were considered trivial, small, moderate, large, and very large, respectively.

Sensitivity of the incremental treadmill test and associated HMPD was assessed using receiving-operator-characteristic (ROC) statistics. The four groups of players were categorized according to their average VL_4_ speed, and the dichotomization cut-off was determined using groups’ mean value + smallest worthwhile change (SWC). SWC between means was assumed as 0.2 × SD [22]. An AUC > 0.70 and the lower CI > 0.50 was classified as a “good” point of reference [18]. All ROC curve results were shown as AUC ± 95% CI. Moreover, the Youden index (YI) was calculated from all ROC curve plots and the maximum value of the index used as a criterion for selecting the optimum cut-off point from each HMPD marker to discriminate a player with high aerobic fitness level or low aerobic fitness level [24]. The YI ranged between 0 and 1, where 0 indicates that a test reflects no diagnostic value and 1 indicates perfect discriminatory value [25]. The alpha level of significance was set at 0.05. All data were analysed using SPSS Statistics version 22 for Windows (IBM Corp., Armonk, IL, USA).

## RESULTS

Analysis of the 1062 individual official matches showed that soccer players covered, on average, 1990 ± 386 m at HMPD (> 25 W·kg^-1^) (range: 1175–2800 m). This corresponds to 18.6% of the total match distance. Details of match activities and physiological data are presented in Table 1A. Significant group differences were found for physiological and metabolic power metrics (Table 1A and Table 1B). The association between VL_4_ and metabolic power metrics variables is reported in Table 2. Large to very large correlations were found between VL_4_ and all metabolic power metrics match variables (Table 2). ROC showed that values of VL_4_ were sensitive in detecting HMPD variations in full back, midfielder and forward groups of players, as indicated by the area under the curve (central back .78, 95%CI .47 to .95; full back .93, 95%CI .64 to 0.99; midfielder .88, 95%CI .67 to 0.98; forward .90, 95%CI .62 to 0.99). The resulting cut-offs for HMPD were: > 1450 m for central back, > 1990 m for full back, > 2170 m for midfielder and > 1670 m for forward groups, respectively (Table 3).

**TABLE 1B:** Effect size of the differences.

Variables	CB vs FB	CB vs MF	CB vs FW	FB vs MF	FB vs FW	MF vs FW
**Max heart rate (bpm)**	0.26 (Moderate)	0.21 (Moderate)	0.34 (Moderate)	0.08 (Trivial)	0.07 (Trivial)	0.17 (Small)
**Max lactate post treadmill test (mml·L^-1^)**	0.11 (Small)	0.03 (Trivial)	0.16 (Small)	0.09 (Trivial)	0.28 (Moderate)	0.22 (Moderate)
**Velocity at 4 mmol·L^-1^ (km·h^-1^)**	0.29 (Moderate)	0.64 (Large)	0.19 (Small)	0.31 (Moderate)	0.10 (Small)	0.44 (Moderate)
**Totale distance (m)**	0.45 (Moderate)	0.92 (Very large)	0.12 (Small)	0.41 (Moderate)	0.34 (Moderate)	0.81 (Very large)
**Anaerobic index (%)**	0.04 (Trivial)	0.15 (Small)	0.02 (Trivial)	0.19 (Moderate)	0.06 (Trivial)	0.13 (Small)
**Equivalent distance (%)**	0.18 (Small)	0.19 (Small)	0.14 (Small)	0.01 (Trivial)	0.04 (Trivial)	0.04 (Trivial)
**Distance > 25 Watt·Kg^-1^ (m)**	0.63 (Large)	0.98 (Very large)	0.37 (Moderate)	0.28 (Moderate)	0.26 (Moderate)	0.58 (Large)
**Power Events (number)**	0.22 (Moderate)	0.87 (Very large)	0.14 (Small)	0.62 (Large)	0.08 (Trivial)	0.73 (Large)
**Power events recovery power (Watt·Kg^-1^)**	0.16 (Small)	0.46 (Moderate)	0.12 (Small)	0.28 (Moderate)	0.28 (Moderate)	0.61 (Large)
**Power events recovery time (s)**	0.10 (Small)	0.51 (Large)	0.10 (Small)	0.40 (Moderate)	0.00 (Trivial)	0.40 (Moderate)
**Power events average time (s)**	0.12 (Small)	0.05 (Trivial)	0.00 (Trivial)	0.08 (Trivial)	0.14 (Small)	0.06 (Trivial)
**Power events average power (Watt·Kg^-1^)**	0. 19 (Small)	0.07 (Trivial)	0.34 (Moderate)	0.13 (Small)	0.14 (Small)	0.30 (Moderate)

**Table 2:** Correlation matrix of the resulting associations among velocity at 4mmol·L^-1^ and the metabolic power metrics considered.

Position	Distance >25 Watt·Kg^-1^ (m)	Power Events (number)	Power events recovery power (Watt·Kg^-1^)	Power events recovery time (s)
Centralback (n = 13)	0.46 (-0.11 to 0.80)	0.41 (-0.15 to 0.77)	0.32 (-0.27 to 0.74)	-0.34 (-0.75 to 0.26)
Fullback (n = 13)	0.75^#^ (0.34 to 0.92)	0.64^§^ (0.14 to 0.88)	0.71^#^ (0.26 to 0.91)	-0.59^§^ (-0.86 to -0.07)
Midfielder (n = 22)	0.74^†^ (0.46 to 0.88)	0.63^#^ (0.29 to 0.83)	0.66^†^ (0.34 to 0.85)	-0.62^#^ (-0.82 to -0.26)
Forward (n = 14)	0.89^†^ (0.67 to 0.96)	0.70^#^ (0.27 to 0.90)	0.75^#^ (0.37 to 0.92)	-0.76^#^ (-0.92 to -0.37)
All players (62)	0.83^†^ (0.72 to 0.89)	0.71^†^ (0.56 to 0.81)	0.72^†^ (0.57 to 0.82)	-0.70^†^ (-0.81 to -0.55)

Note: Data are reported as coefficient of correlation and 95% confidence intervals;

§ p < 0.05;

# p < 0.01;

† p < 0.001.

**Table 3:** Area Under the Curve (AUC) and 95% CI from the Receiving Operator Characteristic curve (ROC) analysis and the Youden Index (YJ) according to playing position.

Position	AUC	95% CI	Cut-off HMPD (m)	YJ
**Central back (n = 13)**	0.78	.47 to 0.95	> 1450	0.55
**Full back (n = 13)**	0.93	.64 to 0.99	> 1990	0.88
**Midfielder (n = 22)**	0.88	.67 to 0.98	> 2170	0.72
**Forward (n = 14)**	0.90	.62 to 0.99	> 1670	0.86

## DISCUSSION

The main finding of this study is that, supporting our hypothesis, the aerobic fitness calculated during the incremental test (velocity at VL_4_) is significantly associated with metabolic power metrics considered in this study (i.e., number of power events and recovery time between power events, Table 2) in elite soccer players.

Despite the different player tracking technology used, the total distance reported in the present study is similar to that recently reported in top-level soccer players irrespective of their playing position [6, 10]. Our study extends these findings, in that we assessed, for the first time, the metabolic power metrics during official matches taking into consideration the position of players on the field. This approach revealed that central backs covered less high-metabolic power distance and performed fewer power events than players in the other field positions, a finding likely linked to the tactical role. In line with this reasoning, midfielders covered a considerable distance at a high metabolic power as well as the largest number of power events compared to players in the other playing positions. In addition, midfielders had greater power recovery than central backs and forwards and less recovery time after power events than central back, full back, and forward players (Table 1A).

These differences in physical and physiological data may be explained by the need of the midfielders to maintain the best team’s spatial structure, based on relative player tactical and physical performance capacity, during actual match play.

In recent years, many studies have investigated aerobic fitness and its influence on soccer match physical parameters but only considered the speed category approach [12, 26]. This approach that uses running speed alone underestimates the high-intensity demands of a soccer match [6, 8]. It does appear that measurements of metabolic power metrics would better inform us about the true physiological efforts experienced during actual match play.

In this study, we found significant relationships between velocity at VL_4_ and magnitude of power events. This association suggests that the ability to produce high intensity bouts during a match is associated with a higher aerobic fitness level.

To establish the effectiveness of aerobic fitness to better maintain the team’s spatial structure, we examined relationship between VL_4_ speed and: 1) the average power recovery after high-intensity bouts and 2) the recovery time between power events. The data showed a strong positive relation between aerobic fitness and repeated high-intensity sequences (number of power events, average power events’ recovery power and average power events’ recovery time), as indicated by the magnitude of correlation, ranging from large to very large. This finding reinforces the relevance of aerobic training in soccer players.

Previous data showed that high intensity in soccer matches is also the result of the tactical playing strategy of the team [27]. Players who play in roles where running is more fundamental almost always succeed in expressing high physical performance values [28, 29]. Discriminating the effects of physiological performance from the effects of performance linked to the specific role of the player would allow us to gain more information on the physical potential of each player. From our data, the individual differences in HMPD and in power events were related not only to field position but, interestingly, also to the velocity reached at VL_4_. In fact, significant positive relationships between VL_4_ speed and metabolic power metrics were found even in the same position groups (Table 2). These individual differences in physiological and physical performance should be taken into account when planning the training and match strategy.

The only group showing a poor correlation between aerobic fitness and metabolic power metrics was that of central backs. It could be argued that the lack of association between aerobic fitness and metabolic variables is due to the lower demand of the game for them, which determines lower interindividual variability in the kinematic and physiological demand [10].

In this study, the sensitivity of the incremental test was assessed using ROC curves and was made by dichotomizing the sample size using the SWC in the mean. The rationale of this analysis assumed that players were at an aerobic fitness level above mean + SWC. Briefly, the hypothesized SWC expected as an outcome of a training intervention could discriminate players with a higher level of aerobic fitness than the rest of their own group [30]. The ROC analysis showed that VL_4_ speed was a sensitive test measure for detecting aerobic fitness in full backs, midfielders and forward players (Table 3). The resulting ROC-curve analysis cut-off value for HMPD was > 2167 m for midfielders, > 1991 m for full backs, and > 1667 m for forwards. Consequently, soccer coaches and strength conditioning trainers, when using the HMPD with elite soccer players, may regard players having HMPD equal to or higher than > 2170 m, > 1990 m and > 1670 m as possessing superior aerobic fitness status. This optimal cut-off value was represented by the higher Youden index (sensitivity + specificity – 1), which represents a function of maximized sensitivity and specificity with respect to an optimal cut-point. Therefore, the Youden index provides an immediate measure of the maximum overall correct classification rate for a given marker.

These notions are relevant to improving our understanding of the association between aerobic fitness and physical soccer performance, whereas studying running speed assessments alone renders impossible an analysis of relevant parameters such as acceleration and deceleration.

Given the large number of professional soccer players and official matches analysed in this study, we can suppose that the results presented here reveal a high level of aerobic fitness and metabolic power metrics data for soccer players and that the study results may be generalized to players competing at the same elite level. Consequently, the external validity of the findings ensuing from this study should be warranted [31].

The main limitation of this study is the use of a convenience sample (i.e., team and club study), and consequently this investigation should be regarded as a case study. However, the provided confirmation of results reported in previously published team studies conducted over the same elite professional soccer players may suggest the validity of the used observational design [10, 13].

In conclusion, this study provided further evidence that submaximal aerobic fitness positively affects match physical performance in elite male soccer players [14]. In fact, interindividual aerobic fitness profiles are mainly associated with high anaerobic metabolic match metrics and suggest that training at high intensity is mandatory in developing maximal and submaximal aerobic fitness [6, 10, 15]. The association of aerobic fitness with metabolic power supports the use of player tracking technology for the assessment of external load during matches and training in soccer players. Given the advances in portable match analysis devices, metabolic power metrics may be easily assessed and could help guide coaches to quantify and regulate the individual training load [6, 10].

Further studies addressing different aspects of fitness and match metabolic power metrics in professional soccer with this approach are warranted (i.e., longitudinal validity).
